# A prospective study on the precision of height data from electronic medical records in tidal volume calculation for lung-protective ventilation

**DOI:** 10.1097/MD.0000000000036196

**Published:** 2023-11-24

**Authors:** Salman Mohamed, Kavita Batra, Nicole Pang, Elliot Runge, Mutsumi John Kioka

**Affiliations:** a Kirk Kekorian School of Medicine at University of Nevada, Las Vegas, NV; b Office of Research, Kirk Kerkorian School of Medicine at University of Nevada, Las Vegas, NV; c Department of Medical Education and Office of Academic Affairs, Kirk Kerkorian School of Medicine at University of Nevada, Las Vegas, NV; d Division of Pulmonary and Critical Care Medicine, Department of Internal Medicine, Kirk Kerkorian School of Medicine at University of Nevada, Las Vegas, NV.

**Keywords:** acute respiratory failure, height information in the medical record, lung protective ventilation, predicted body weight, tidal volume for the ventilation

## Abstract

Lung-protective ventilation is now the norm for all patients, regardless of the presence of acute respiratory distress syndrome (ARDS), owing to the mortality associated with higher tidal volumes (TV). Clinicians calculate TV using recorded height from medical records and predicted body weight (PBW); however, the accuracy remains uncertain. Our study aimed to validate accurate TV settings for lung-protective ventilation by examining the correlation between the charted height and bedside measurements. In a single-center study, we compared PBW-based TV calculated from recorded height to PBW-based TV from measured height and identified factors causing height overestimation during charting. Our team measured patient height within 24 hours of admission using metal tape. TV calculated from recorded height (6–8 mL/kg PBW) was significantly larger (391.55 ± 65.98 to 522.07 ± 87.97) than measured height-based TV (162.62 ± 12.62 to 470.28 ± 89.64) (*P* < .01). In the height overestimated group, 57.7% were prescribed TV by healthcare provider, which was more than TV of 8 mL/kg of PBW, as determined by measured height. Negative predictors for height overestimation were male sex (OR: 0.45 [95% CI: 0.25–0.82]; *P* = .008) and presence of driver’s license information (OR: 0.45 [95% CI: 0.25–0.80]; *P* = .007), whereas Asian ethnicity was a positive predictor (OR: 4.34 [95% CI: 1.09–17.27]; *P* = .04). The height overestimation group had a higher in-patient mortality rate (38.5%) than the matched/underestimation group (20%) (*P* < .01). In stadiometer-limited hospitals, the PBW-based TV is overestimated using the recorded height instead of the measured height. In the group where heights were overestimated, over half of the patients received TV prescriptions from healthcare providers that surpassed the TV of calculated 8 mL/kg PBW based on their measured height. The risk factors for height overestimation include female sex, Asian ethnicity, and missing driver’s license data. Alternative height measurement methods should be explored to ensure precise ventilation settings and patient safety.

## 1. Introduction

Lung protective ventilation has become a globally accepted strategy for managing patients with acute respiratory distress syndrome (ARDS).^[1]^ In the past, clinicians commonly employed large tidal volumes when utilizing mechanical ventilators. The traditional tidal volume (TV) ranged from 10 to 15 mL per kilogram (kg) of predicted body weight (PBW).^[2]^ However, in the 1990s, the National Heart, Lung, and Blood Institute in the United States conducted an Acute Respiratory Distress Syndrome Network study, which demonstrated that a low-tidal volume strategy significantly reduced mortality and improved patient outcomes.^[2]^ Additional evidence has indicated that lung protective ventilation may also benefit patients without ARDS by lowering inflammation and lung injury.^[3]^ In a national randomized controlled trial conducted in 2015, it was found that using a low tidal volume of 4 to 6 mL/kg PBW at the initiation of ventilation in intensive care unit (ICU) patients without ARDS yielded significant results compared to a high tidal volume ventilation strategy using TV from 8 to 10 mL/kg PBW.^[4]^ The current guideline for mechanical ventilation, the ARDS Network Protocol, recommends calculating the initial TV using PBW derived from the patient’s height, with PBW multiplied by 6 to 8 mL/kg.^[2]^ Presently, modern intensivist relies on the documented height in the patient’s chart to determine the initial TV setting for patients, irrespective of whether they have ARDS. The recorded height in the chart often originates from self-reporting by the patient or their family, information from a driver’s license, or sometimes an estimation made by a triage nurse in the emergency department (ED) upon admission.^[5]^

However, it remains uncertain whether the documented height from the chart accurately corresponds to the PBW used to calculate the TV for lung protective ventilation in intubated patients. This study was conducted to investigate the correlation between the recorded height in the chart and bedside measurements, with a specific focus on the accurate calculation of the tidal volume for current recommended lung-protective ventilation. Additionally, we examined the factors associated with height overestimation, as it can potentially pose a risk of lung damage through overexpansion when using a mechanical ventilator. Furthermore, we assessed the effect of height overestimation on patient clinical outcomes.

## 2. Methods

### 2.1. Study design, setting, and population

This study was approved by the Institutional Review Board of the University of Nevada Las Vegas, Nevada (IRB No 1203933-2) and the University Medical Center of Southern Nevada, Las Vegas, Nevada (IRB No UMC-2017-115).

**Figure 1.**
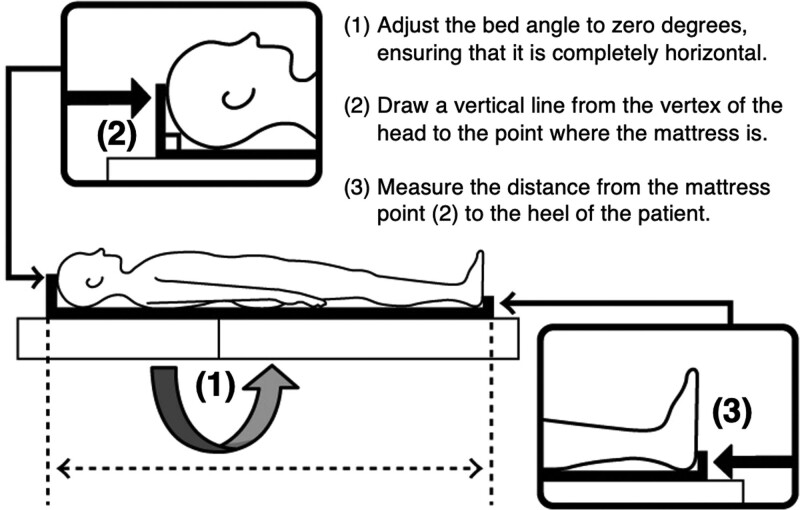
Method of bedside height measurement diagram.

We conducted a prospective analysis using data collected from an urban-based tertiary care teaching hospital between May 22, 2018, and August 24, 2021. The study enrolled patients aged ≧ 18 years admitted to the ICU with a primary diagnosis of acute respiratory failure. Nevertheless, due to the intermittent availability of the investigator team, not all patients underwent screening. The Medical Intensive Care Unit (MICU) comprises 19 beds, and the ICU is staffed by internal medicine residents, pulmonary/critical care medicine fellows, and attending physicians who provide care during and after admission. The investigator teams visited the patient’s bedside within the first 24 hours of the admission. First, the bed angle was adjusted to zero degrees to flatten it, and then the patient’s body was straightened as much as possible. Next, a vertical line was drawn from the patient’s vertex to the mattress, and the distance from that point on the mattress to the patient’s heel was measured using a metal tape (Fig. 1). To calculate the PBW, we employed the following equations:^[2]^

PBW (males) = 50 + 0.91 [height (cm) − 152.4] in kg.

PBW (females) = 45.5 + 0.91 [height (cm) − 152.4] in kg.

In our hospital, the ED does not utilize a stadiometer or height gauge to measure the height of patients. Sources of recorded height in electronic medical records (EMR) include self-reporting by the patient, information from driver’s licenses, and estimations made by triage nurses in the ED during admission. For this study, we defined the recorded height as the average value between the height documented on the first page of the EMR and the height recorded in the driver’s license information on the face sheet, if available.

**Figure 2.**
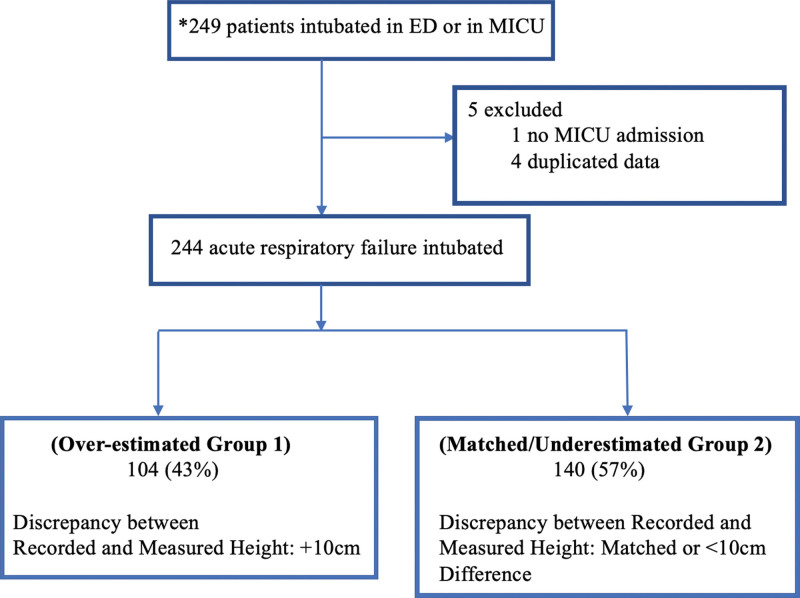
Cohort study flowchart. ED = emergency department, EMR = electronic medical record, MICU = medical intensive care unit. *During the study period, over 249 patients were admitted to the MICU. However, due to the transition from paper charts to EMR, it was not possible to retrospectively obtain the complete set of admission data.

The patients were divided into 2 groups based on the comparison between their average recorded height and measured heights. Group 1, referred to as the “Overestimated Group,” consisted of patients whose documented height exceeded the measured height by > 10 cm. Group 2, known as the “Matched/Underestimated Group,” included patients whose recorded height either had a difference smaller than 10 cm or was smaller than the measured height itself (Fig. 2). We chose to define overestimation as exceeding 10 cm because, despite our careful bedside measurement method, a margin of error of less than 10 cm could still occur. Additionally, to avoid any influence on the triage nurse’s height estimation process, the nurse was unaware that the investigator team measured the patient’s height during the admission procedure.

### 2.2. Statistical analysis

The sample size calculation for this study was as follows. Before commencing data collection, 20 of a subgroup of participants were selected, and their tidal volume (TV) of 8 mL/kg of PBW was determined using measured height and documented height. The analysis revealed a mean difference of 52.56 standard deviations (SD) = 87.31 between the 2 groups. Based on this preliminary analysis, multiple scenarios were evaluated to determine the sample size required to detect differences with a specified effect size, using an alpha level of 0.05. It was determined that a sample size of 170 would be sufficient to detect a 25% difference, 266 for a 20% difference, and 472 for a 15% difference. Considering these findings from the preliminary sample data, a sample size of n = 250 was deemed appropriate to detect an approximate 20% difference in the TV of PBW between the measured height and documented height groups (Table, Supplemental Digital Content, http://links.lww.com/MD/K784).

First, univariate and bivariate tests were conducted to analyze the data. All assumptions, including normality and homogeneity of variance, were assessed. Categorical variables are represented as frequencies and proportions, whereas continuous variables are represented as means and standard deviations. Groups were categorized as patients whose height was overestimated by > 10 cm from the measured height (Group 1) versus patients whose height was matched or less than the measured height (Group 2). The Chi-square/Fisher exact test was used to compare the nominal groups. Continuous outcomes were compared using independent samples (*t* test or Welch’s *t* test) if homogeneity of variance was not assumed. For non-normally distributed continuous data, we applied square root or cube root transformations to approximate skewed variables or those with negative values to the normal approximation. The transformed variables were back-transformed for interpretation after the analysis. Logistic regression was performed to ascertain the effects of the selected independent variables on the likelihood of overestimating the height. The selection of the variables was made based on theoretical, conceptual, and statistical grounds. Estimates for the parameters were obtained using the maximum likelihood estimation method with 95% Wald confidence limits for the logistic model. The final model was selected based on the Akaike information criterion and The Schwarz criterion (SC).^[6]^ The significance level was set at *P* < .05. The normal approximation of the binomial distribution method was used to calculate the 95% confidence intervals of the proportions in the univariate analyses. All analyses were conducted using IBM SPSS version 27 and SAS 9.4.

## 3. Results

Throughout the study duration, the MICU beds saw the admission of over 249 patients primarily diagnosed with acute respiratory failure. Within the initial 24 hours of admission, 249 patients were screened, considering the intermittent availability of the investigator team. Out of the complete pool of admissions, 249 patients underwent assessment by the investigator team. A slight refinement was implemented, resulting in the exclusion of 5 patients due to either duplicated data or their admission occurring outside the ICU setting (Fig. 2). Among the remaining 244 patients who were intubated during admission, they fell into the following categories based on height estimation:104 patients (43%) had an overestimated height, 140 patients (57%) had a height that was matched/underestimated or had a difference of less than 10cm compared to the measured height (Fig. 2).

Baseline Characteristics of the study population are presented in Table 1.

**Table 1:** Demographic Characteristics.

		(N = 244)	(95% CI)
Age (yr.)		58.97 ± 15.5	(57.01–60.92)
Male sex – no. (% [95% CI])		153 (62.7 [56.3–68.7])	
Race/Ethnicity – no. (% [95% CI])	White	134 (54.9 [48.4–61.3])	
	African American	50 (20.5 [15.3–26.1])	
	Hispanic	37 (15.2 [10.9–20.3])	
	Asian	13 (5.3 [2.8–8.9])	
	Other	10 (4.1 [1.9–7.4])	
BMI on the continuous scale in kg/m^2^		30.36 ± 9.91	(29.10–31.61)
BMI categories (in kg/m^2^) – no. (% [95% CI]) (Underweight)	<18.5	8 (3.3 [1.4–6.4])	
(Healthy weight)	18.5–24.9	75 (30.7 [25.0–36.9])	
(Overweight)	25–29.9	62 (25.4 [20.0–31.3])	
(Obese)	>30	99 (40.6 [34.3–47.0])	
Clinical characteristics			
Charlson comorbidity index (0–24 points)		3.98 ± 3.2	(3.57–4.39)
Preexisting conditions – no. (% [95% CI])			
ARDS		49 (20.1 [15.2–25.6])	
Arrhythmia		2 (0.8 [0.1–2.9])	
Cerebral vascular accident		13 (5.3 [2.8–8.9])	
Connective tissue disease		0 (0.0 [0.00–1.5])	
Coronary artery disease		25 (10.2 [6.7–14.7])	
Congestive heart failure		22 (9.0 [5.7–13.3])	
Chronic kidney disease		33 (13.5 [9.5–18.5])	
Chronic obstructive lung disease		31 (12.7 [8.8–17.5])	
Dementia		5 (2.0 [0.6–4.7])	
Diabetes		78 (32.0 [26.1–38.2])	
Hemiplegia		0 (0.0 [0.00–1.5])	
HIV		4 (1.6 [0.5–4.1])	
Hypertension		124 (50.8 [44.3–57.3])	
Metastatic disease		23 (9.4 [6.1–13.8])	
Myocardial infarction		8 (3.3 [1.4–6.4])	
Leukemia/lymphoma		0 (0.0 [0.00–1.5])	
Liver disease		11 (4.5 [2.2–7.9])	
Peptic ulcer disease		7 (2.9 [1.1–5.8])	
Peripheral vascular disease		9 (3.7 [1.7–6.8])	
Solid tumor		4 (1.6 [0.5–4.1])	

Plus-minus values are means ± SD.

Driver license available = 153(62.7%).

ARDS = acute respiratory distress syndrome, BMI = body mass index, CI = confidence interval, HIV = human immunodeficiency virus, SD = standard deviation.

The mean [±standard deviation: SD] age of the study population was 58.97 ± 15.5 (95% CI: 57.01–60.92). Of the participants, 153 (62.7% [95% CI: 56.3–68.7]) were male. More than 40 percent of the patients who had a body mass index (BMI) > 30 kg/m^2^ were categorized as obese. The mean ± SD of Charlson comorbidity index^[7]^ was 3.98 ± 3.2 (95% CI: 3.57–4.39), indicating a moderate comorbidity burden in the population. Among the preexisting conditions, 49/244 individuals (20.1% [95% CI: 15.2–25.6]) were diagnosed with ARDS. In the case of 153/244 patients (62.7%), driver’s license information was available during admission to the hospital.

The characteristics of the study groups are summarized in Table 2. No significant differences were observed between the groups in terms of Race/Ethnicity or BMI. However, Group 1 showed a higher mean age [±standard deviation: SD] of (61.37 ± 15.28) than Group 2 (57.17 ± 15.4) (*P* = .04). Additionally, Group 2 had a higher percentage of males (70.0%) than Group 1 (52.9%) (*P* = .006). The Charlson comorbidity index was also higher in Group 1 (4.49 ± 3.48) than in Group 2 (3.64 ± 3.00) (*P* = .04). Furthermore, the prevalence of preexisting conditions such as chronic kidney disease, chronic obstructive lung disease, and peripheral vascular disease was higher in Group 1 than in Group 2.

**Table 2:** Demographic Characteristics of Study groups.

		Group 1	Group 2	*P* value
		Overestimated	Matched/underestimated	
		(N = 104)	(N = 140)	
Age		61.37 ± 15.28	57.17 ± 15.4	.04
Male sex – no. (%)		55 (52.9)	98 (70.0)	.006
Race/ethnicity – no. (%)	White	54 (51.9)	80 (57.1)	.2
	African American	19 (18.3)	31 (22.1)	
	Hispanic	16 (15.4)	21 (15.0)	
	Asian	9 (8.7)	4 (2.9)	
	Other	6 (5.8)	4 (2.9)	
BMI on the continuous scale (kg/m^2^)		30.84 ± 10.98	29.94 ± 9.05	.5
BMI categories in kg/m^2^ – no. (%)	<18.5 (Underweight)	2 (1.9)	6 (4.3)	.5
	18.5–24.9 (Healthy weight)	31 (29.8)	44 (31.4)	
	25–29.9 (Overweight)	31 (29.8)	31 (22.1)	
	>30 (Obese)	40 (38.5)	59 (42.1)	
Clinical characteristics				
Charlson comorbidity index (0–24 points)		4.49 ± 3.48	3.64 ± 3.00	.04
Preexisting conditions – no. (%)				
ARDS		24 (23.3)	25 (17.9)	.3
Arrhythmia		2 (1.9)	0 (0.0)	.2
Cerebral vascular accident		6 (5.8)	7 (5.0)	.8
Coronary artery disease		11 (10.6)	14 (10.0)	.8
Congestive heart failure		11 (10.6)	11 (7.9)	.5
Chronic kidney disease		21 (20.2)	12 (8.6)	.009
Chronic obstructive lung disease		20 (19.2)	11 (7.9)	.007
Dementia		1 (1.0)	4 (2.9)	.3
Diabetes		38 (36.5)	40 (28.6)	.1
HIV		1(1.0)	3 (2.1)	.4
Hypertension		51 (49.0)	73 (52.1)	.6
Metastatic disease		13 (12.5)	10 (7.1)	.2
Myocardial infarction		2 (1.9)	6 (4.3)	.3
Liver disease		4 (3.8)	7 (5.0)	.7
Peptic ulcer disease		1 (1.0)	6 (4.3)	.2
Peripheral vascular disease		8 (7.7)	1 (0.7)	.005
Solid tumor		1 (1.0)	3 (2.1)	.5

Plus-minus values are means ± SD.

ARDS = acute respiratory distress syndrome, BMI = body mass index, HIV = human immunodeficiency virus, SD = standard deviation.

A comparison between the expected TV based on the recorded height and measured height is presented in Table 3. The mean [±standard deviation: (SD)] of calculated TV using recorded height (calculated as PBW times 6–8 mL/kg) resulted in significantly larger values from 391.55 ± 65.98 to 522.07 ± 87.97 compared to those obtained using measured height-based TV (calculated as PBW times 6–8 mL/kg) from 162.62 ± 12.62 to 470.28 ± 89.64 (*P* < .001).

**Table 3:** Expected tidal volume by paired *t* test.

Tidal volume (mL/kg of PBW)	Tidal volume of PBW (recorded height)*	Tidal volume of PBW (measured height)†	*P* value
6 (mL/kg) × PBW	391.55 ± 65.98	352.71 ± 67.23	<.001
8 (mL/kg) × PBW	522.07 ± 87.97	470.28 ± 89.64	<.001

Plus-minus values are means ± SD.

(a)Mean ± SD of current TV setting by provider care team at the time of evaluation by investigator team: 443.3 ± 35.58.

(b) The mean increase in the current TV setting by healthcare provider compared to TV calculated at 8 mL/kg of PBW using measured height is 76.19 mL (95% CI: 58.43–93.95).

(c)Overestimated (Group 1): 60/104 (57.7%) were prescribed current TV greater than TV of 8 mL/kg of PBW based on measured height. Conversely, Matched/Underestimated (Group 2): 29/140 (20.7%) were prescribed current TV greater than TV of 8 mL/kg of PBW based on measured height.

CI = confidence interval, PBW = predicted body weight, SD = standard deviation.

* PBW based on recorded height.

† PBW based on measured height.

The mean ± SD of the current TV setting by the healthcare provider at the time of evaluation by the investigator team was 443.3 ± 35.58. Furthermore, the average disparity between TV calculated at 8 mL/kg of PBW using the measured height and the current prescribed TV setting by healthcare providers amounted to 76.19 mL (95% CI: 58.43–93.95).

Within the subgroup of overestimated cases (Group 1), out of a total of 104 cases, 60 (57.7%) had been prescribed a current TV greater than TV of 8 mL/kg of PBW based on their measured height. Conversely, Matched/Underestimated group (Group 2), out of a total of 140 cases, 29 (20.7%) were prescribed current TV greater than TV of 8 mL/kg of PBW based on measured height. These findings have been detailed in the footnote of Table 3.

The significant predictors of overestimation in the charts are presented in Table 4. Negative predictors of overestimation of height were male sex (OR: 0.45 [95% CI:0.25-0.82]; *P* = .008) and driver’s license availability (OR: 0.45 [95% CI:0.25-0.80]; *P* = .007). A positive predictor of overestimation of height was Asian ethnicity (OR: 4.34 [95% CI:1.09-17.27]; *P* = .04).

**Table 4:** Logistic regression predicting likelihood of height overestimation among 241 patients admitted to an intensive care unit.

Variable	OR (95% CI)	*P* value
Age	1.01 (0.98–1.03)	.7
Sex, male	0.45 (0.25–0.82)	.008
Race or ethnic group		
White*	1.08 (0.51–2.26)	.8
Hispanic*	1.24 (0.49–3.17)	.6
Asian*	4.34 (1.09–17.27)	.04
Other*	2.05 (0.47–8.97)	.3
Driver license	0.45 (0.25–0.80)	.007
CKD	2.18 (0.896–5.29)	.086
COPD	2.28 (0.95–5.48)	.065
PVD	8.01 (0.88–72.74)	.064

CI = confidence interval, CKD = chronic kidney disease, COPD = chronic obstructive lung disease, OR = odds ratio, PVD = peripheral vascular disease.

* ORs were calculated in comparison to the reference group of Black individuals.

The main clinical outcomes are summarized in Table 5. Group 1 exhibited a significantly higher mortality rate of 40/104 (38.5%) than Group 2, with a mortality rate of 28/140 (20%) (*P* = .001). However, no significant differences were observed between the groups in terms of healthcare resource utilization, including ICU days, ventilator days, and hospital days. Additionally, the ventilator parameters during the first 24 hours were also not found to be significantly different.

**Table 5:** Main outcome.

Variables	Group 1	Group 2	*P* value
	Overestimated	Matched/underestimated	
	(N = 104)	(N = 140)	
	No./total no. (%)		
Clinical outcomes			
In-patient mortality	40/104 (38.5)	28/140 (20.0)	.001
Healthcare resource utilization			
ICU days	7.72 ± 1.29	7.08 ± 1.29	.4
Ventilator days	7.84 ± 3.24	6.76 ± 2.13	.3
Hospital days	14.59 ± 3.24	13.83 ± 2.01	.6
Ventilator parameters			
Peak pressure (cm of water)	23.37 ± 8.13	24.43 ± 9.18	.3
Plateau pressure (cm of water)	17.36 ± 6.42	18.16 ± 6.75	.4
Driving pressure (cm of water)	11.71 ± 6.75	12.20 ± 7.37	.6
Driving pressure > 15 (cm of water)	25/104 (26.6)	40/140 (32.3)	.4
PaO2:FiO2	236.54 ± 138.18	275.84 ± 266.60	.2
PEEP (cm of water)	6.22 ± 3.03	5.96 ± 2.24	.4

Plus-minus values are means ± SD.

FiO2 = fraction of inspired oxygen, ICU = intensive care unit, PaO2 = partial pressure of arterial oxygen, PBW = predicted body weight, PEEP = positive end-expiratory pressure, SD = Standard Deviation, TV = tidal volume.

## 4. Discussion

Our study findings emphasize the impact of using the recorded height in the EMR chart for calculating the PBW in lung-protective ventilation. This approach yielded an average TV based on recorded heights ranging from 6 to 8 mL/kg, which was significantly higher than the values obtained when employing the measured height for TV calculations. This comparison highlights a substantial difference between the 2 approaches in determining the appropriate TV levels for achieving lung-protective ventilation (Table 3). The overestimated group (Group 1) had over half of the patients receiving a tidal volume (TV) from the provider team that exceeded 8cc/kg of predicted body weight (PBW), as determined by measured height. In comparison, the Matched/Underestimated group (Group 2) comprised only one-fifth of the patients who were administered a TV exceeding 8 cc/kg PBW based on their measured height.

Several factors contribute to the occurrence of overestimated height recorded in the chart, including sex (female), Asian race, and the absence of driver’s license information (Table 4). Furthermore, the group with overestimated height (Group 1) demonstrated a higher in-house mortality rate than the matched/underestimated height group (Group 2). (Table 5).

Accurate height measurement plays a critical role in modern medical care. It is not only important in ventilator settings but also in determining proper medication dosages, conducting a nutritional assessment, and monitoring disease progression.^[8,9]^ The current recommended guidelines for clinical height measurement emphasize the use of stadiometers, as the accuracy of this measure directly contributes to improved medical care.^[10]^

Our data suggest that if the height recorded in a patient’s chart is solely used to calculate PBW for TV settings, clinicians may inadvertently prescribe TV values higher than appropriate based on the measured height. This finding was supported by a previous study in which height records in EMRs were frequently reliant on patient self-reporting or visual estimates, which can introduce inaccuracies in the measurement.^[5]^

The average [±SD] of the current TV setting, as determined by the provider team during the investigation, was 443.3 ± 35.58. The average rise in the present TV configuration, in contrast to the TV computed at 8 mL/kg of PBW using measured height, is 76.19 mL. Furthermore, among the subset of instances characterized by overestimation (Group 1), a larger number of patients were assigned a TV exceeding the 8 mL/kg PBW based on their measured height when compared to the Matched/Underestimated group (Group 2). However, the extent to which this TV set is influenced by inaccuracies in height records or noncompliance with lung-protective ventilation strategies remains uncertain.

Several studies have been published regarding the noncompliance rate of lung protective ventilation in North America. One study reported an overall adherence rate of 33.7% to lung protective ventilation.^[11]^ As for the degree of compliance to lung protective ventilation in our hospital, it remains uncertain as we have not conducted a survey among physicians to assess their compliance with the current recommendation for lung protective ventilation.

Extensive research has been conducted to identify the risk factors associated with prescribing high tidal volumes in ventilated patients, including obesity, female sex, and non-adherence to lung-protective ventilation strategies.^[11–13]^ In our study, BMI was not incorporated as a variable for obesity in the logistic regression model for predicting overestimated height, as our hypothesis primarily focused on the inaccuracies of recorded height in the chart and BMI may also be prone to inaccuracies. Our study model revealed that the risk factors for height overestimation were female sex, Asian race, and the absence of driver’s license information. Previous research on children has shown that short stature may lead to height overestimation by both themselves and their parents.^[14]^ The overestimation observed in female and Asian race groups can be partially attributed to the fact that, on average, females tend to have a shorter stature than male. Significant variation exists within and between populations; Asian individuals, in particular, generally exhibit a shorter stature than other racial or ethnic groups.^[14]^ These factors might contribute to significant errors in height records in females and Asians. However, the extent to which overestimated height records are influenced by triage nurses or self-reported patient information remains unclear.

In North America, individuals typically visit the Department of Motor Vehicles to acquire driver’s licenses. One study demonstrated inaccuracies in height information found in driver’s licenses, indicating potential discrepancies.^[15]^ However, our study showed that the absence of driver’s license information is a risk factor for height overestimation; the exact proportion of height information derived from wall rulers and self-reported height in the Department of Motor Vehicles in our patients remains uncertain.

These findings emphasize the potential pitfalls of relying solely on the recorded height in the patient’s chart to calculate the PBW for TV settings in lung-protective ventilation. Clinicians should be cautious and consider incorporating additional factors, such as more accurate measurements or alternative methods for estimating PBW, to ensure appropriate TV settings and minimize the risk of ventilator-associated complications.

Over several decades, numerous clinical studies have consistently demonstrated improved patient outcomes with lung-protective ventilation.^[2]^ According to the prevailing American Thoracic Society Practice Guidelines for Mechanical Ventilation in Adults with ARDS, there appears to be no direct correlation between low tidal volumes and mortality.^[16]^ While it is acknowledged that extremely high TV (ranging from 10–15 mL/kg PBW) is considered the most significant risk factor for developing ventilator-associated lung injury; however, the relationship between ventilation strategies and patient outcomes is multifaceted and may be influenced by various factors, including driving pressure, trans-pulmonary pressure, and individual patient characteristics.^[17–19]^

In the 2015 national randomized study, known as PReVENT, it was observed that even in patients without ARDS, the utilization of a low tidal volume of 4 to 6 mL/kg PBW at the initiation of ventilation resulted in improved outcomes when compared to a high tidal volume ventilation strategy using TV ranging from 8 to 10 mL/kg.^[4]^ Another study demonstrated that an initial TV greater than 8 mL/kg PBW is associated with increased mortality in patients with complicated ARDS.^[20]^

It’s important to highlight that despite the absence of notable deviations in ventilatory parameters between our 2 groups, it should be acknowledged that our assessment took place within the initial 24 hours. As a result, there remains uncertainty regarding whether patients in Group 1 might have been subjected to elevated TV settings and heightened pressures, encompassing factors like plateau pressure and driving pressure, prior to the investigator’s evaluation. This uncertainty arises because the medical team might have adjusted the ventilator settings during the initial period without documenting those changes in the patient’s medical record.

The interpretation of elevated mortality within the overestimated group (Group 1) warrants careful consideration, regardless of the possibility of changing the ventilator setting without documentation. The disparities presented in Table 3 regarding a nominal difference of approximately 50 mL between the recorded and measured heights, equating to a TV discrepancy of 8 mL/kg PBW, are unlikely to have substantially contributed to the observed variance in patient mortality. Additionally, an alternative perspective emerges when considering the demographic distinctions between the 2 groups: Group 1 (the overestimated height group) possessed a notably higher mean age of 61.37 ± 15.28, in contrast to Group 2’s (the matched/underestimated height group) mean age of 57.17 ± 15.4 (*P* = .04). Moreover, Group 1 exhibited a heightened prevalence of preexisting conditions encompassing chronic kidney disease, chronic obstructive lung disease, and peripheral vascular disease. Evidenced by a higher Charlson comorbidity index of 4.49 ± 3.46, Group 1 diverged from Group 2, which demonstrated a comparably lower index of 3.64 ± 3.00 (*P* = .04) as delineated in Table 2. These inherent variations between Group 1 and Group 2 may have undeniably impacted the observed clinical outcomes. As such, the discernible divergence in mortality rates is unlikely to be solely ascribed to dissimilarities in tidal volume administration between the 2 cohorts.

Hence, within the scope of our study, it becomes apparent that the inclination of clinicians to administer higher tidal volumes upon patient admission cannot be exclusively attributed to the recorded height, and establishing a direct causative link to other factors proves to be a complex challenge.

### 4.1. Study limitations

Our study had several limitations that need to be acknowledged. First, it is important to note that this study was conducted at a single center, which may limit the generalizability of our findings to other settings or populations. Our study’s practices and patient characteristics may have been specific to the local context. The prevalence of stadiometer usage during the admission process in hospitals across the United States and worldwide is unknown. Second, we relied on the height recorded in the patient chart and did not directly survey the triage nurses who recorded their height. This makes it challenging to determine the exact source of the recorded height, whether obtained through self-report, triage nurse estimation, or based on the driver’s license information. The accuracy and consistency of the recorded height may vary depending on these factors. Thirdly, due to the nature of our study, there remains uncertainty regarding whether patients might have received different TV settings that were not documented, possibly adjusted by clinicians due to other factors. Fourth, due to limitations in investigator accessibility, not all admitted patients with acute respiratory failure were evaluated. This may introduce selection bias during the screening process. Finally, despite conducting meticulous height measurements at the bedside, it is important to acknowledge that small errors or variations in measurement could still occur. Considering these limitations, caution should be exercised when interpreting and applying our findings.

## 5. Conclusion

In conclusion, in hospital environments where a stadiometer is not used to measure a patient’s height during admission, utilizing the recorded height in the patient’s chart to calculate PBW for TV settings in lung-protective ventilation leads to larger TV values compared to PBW based on measured height, and in the group where heights were overestimated, over half of the patients received TV prescriptions from healthcare providers that surpassed the calculated TV of 8 mL/kg PBW based on their measured height. The risk factors for height overestimation recorded in the patient’s chart included female sex, Asian race, and the absence of driver’s license information. Conducting future research in a more diverse and multicenter setting addressing the limitations of our study would offer valuable insights into this topic.

## Author contribution

**Conceptualization:** Mutsumi John Kioka, Kavita Batra.

**Data curation:** Mutsumi John Kioka, Salman Mohamed, Kavita Batra, Nicole Pang, Elliot Runge.

**Formal analysis:** Kavita Batra.

**Funding acquisition:** Mutsumi John Kioka.

**Investigation:** Mutsumi John Kioka, Salman Mohamed, Kavita Batra, Nicole Pang, Elliot Runge.

**Methodology:** Mutsumi John Kioka, Kavita Batra.

**Project administration:** Mutsumi John Kioka.

**Resources:** Mutsumi John Kioka.

**Software:** Kavita Batra.

**Supervision:** Mutsumi John Kioka, Kavita Batra.

**Validation:** Mutsumi John Kioka, Kavita Batra.

**Visualization:** Mutsumi John Kioka.

**Writing – original draft:** Mutsumi John Kioka, Kavita Batra.

**Writing – review & editing:** Mutsumi John Kioka, Kavita Batra.

## Supplementary Material
