# Supplementary figures and images for: A prospective study on the precision of height data from electronic medical records in tidal volume calculation for lung-protective ventilation
Source: Medicine (Baltimore). 2023 Nov 24;102(47):e36196. doi: 10.1097/MD.0000000000036196 (PMC10681549; doi:10.1097/MD.0000000000036196)

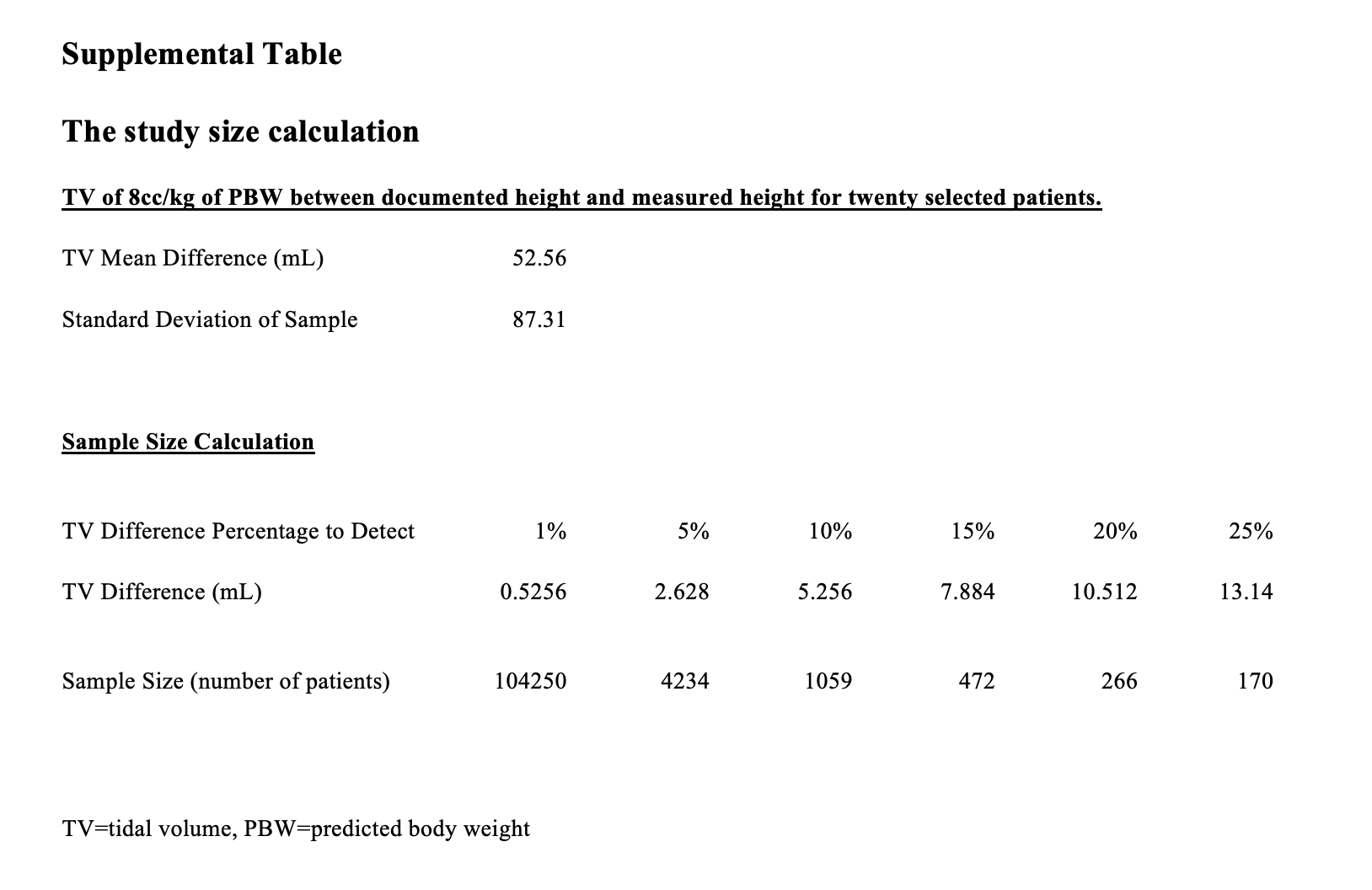

Supplement: Supplementary file 1 [file medi-102-e36196a-s001.docx]
